# Serial SOFA‐score trends in ICU‐admitted COVID‐19 patients as predictor of 28‐day mortality: A prospective cohort study

**DOI:** 10.1002/hsr2.1116

**Published:** 2023-05-02

**Authors:** Farzad Esmaeili Tarki, Siamak Afaghi, Fatemeh Sadat Rahimi, Arda Kiani, Mohammad Varahram, Atefeh Abedini

**Affiliations:** ^1^ Research Department of Internal Medicine Shahid Beheshti University of Medical Sciences Tehran Iran; ^2^ Prevention of Metabolic Disorders Research Center, Research Institute for Endocrine Sciences Shahid Beheshti University of Medical Sciences Tehran Iran; ^3^ Chronic Respiratory Disease Research Center, National Research Institute of Tuberculosis and Lung Diseases, Masih Daneshvari Hospital Shahid Beheshti University of Medical Sciences Tehran Iran; ^4^ Mycobacteriology Research Center, National Research Institute of Tuberculosis and Lung Disease Shahid Beheshti University of Medical Sciences Tehran Iran

**Keywords:** COVID‐19, critical care medicine, infectious diseases, respiratory medicine, SOFA score

## Abstract

**Background and Aim:**

The efficacy of Sequential Organ Failure Assessment (SOFA) score as predictor of clinical outcomes among ICU‐admitted COVID‐19 patients is still controversial. We aimed to assess whether SOFA‐score in different time intervals could predict 28‐day mortality compared with other well‐acknowledged risk factors of COVID‐19 mortality.

**Methods:**

This observational prospective cohort was conducted on 1057 patients from March 2020 to March 2022 at Masih Daneshvari Hospital, Iran. The univariate and multivariate Cox proportional analysis were performed to assess the hazards of SOFA‐score models. Receiver operating characteristic (ROC) curves were designed to estimate the predictive values.

**Results:**

Mean SOFA‐score during first 96 h (HR: 3.82 [CI: 2.75–5.31]), highest SOFA‐score (HR: 2.70 [CI: 1.93–3.78]), and initial SOFA‐score (HR: 1.65 [CI: 1.30–2.11]) had strongest association with 28‐day mortality (*p* < .0001). In contrast, SOFA scores at 48 and 96 h as well as Δ‐SOFA: 48‐0 h and Δ‐SOFA: 96‐0 h did not show significant correlations. Among them, merely mean SOFA‐score (HR: 2.28 [CI: 2.21–3.51]; *p* < .001) remained as independent prognosticator on multivariate regression analysis; though having less odds of predicting value compared with age (HR: 3.81 [CI: 1.98–5.21]), hypertension (HR: 3.11 [CI: 1.26–3.81]), coronary artery disease [CAD] (HR: 2.82 [CI: 1.51–4.8]), and diabetes mellitus (HR: 2.45 [CI: 1.36–2.99]). The area under ROC (AUROC) for mean SOFA‐score (0.77) and highest SOFA‐score (0.71) were larger than other SOFA intervals. Calculating the first 96 h of SOFA trends, it was obtained that fatality rate was <12.3% if the score dropped, between 28.8% and 46.29% if the score remained unchanged, and >50.45% if the score increased.

**Conclusion:**

To predict the 28‐day mortality among ICU‐admitted COVID‐19 patients, mean SOFA upon first 96 h of ICU stay is reliable; while having inadequate accuracy comparing with well‐acknowledged COVID‐19 mortality predictors (age, diabetes mellitus, hypertension, CAD). Notably, increased SOFA levels in the course of first 96 h of ICU‐admission, prognosticate at least 50% fatality regardless of initial SOFA score.

## 1 INTRODUCTION

The burden of severe acute respiratory syndrome coronavirus 2 (SARS‑CoV‑2) infection dramatically has affected the healthcare systems globally since the initiation of the pandemic in December 2019. About 600 million individuals afflicted with coronavirus disease 2019 (COVID‐19) worldwide until the end of July 2022^1^; and it is estimated that 5%–35% of these had experienced to be admitted for at least 1 day in intensive care units (ICUs).^2^ The most common complications causing COVID‐19 patients to be hospitalized include acute respiratory distress syndrome (ARDS),^3^ acute kidney failure,^4^, ^5^ thromboembolic and cardiac events,^6^, ^7^ elevated inflammation conditions,^8^ and liver injuries.^9^ While the severity of COVID‐19 has been started to depreciate by the improvements in the rate of vaccination, the capacity of ICUs could be mostly occupied by COVID‐19 patients^10^; particularly in countries with a slower pace of vaccination, causing them to face consecutive waves of SARS‐CoV‐2 infection.^8^ This issue has created concerns about the capacity of ICUs providing clinical support for patients during the further potential infection waves. This may necessitate health officials to identify patients with lower chance of surviving and to prioritize those requiring to receive mechanical ventilation and other advanced therapeutic options as well.^11^, ^12^ Accordingly, a survey on the comparison of ventilator triage policies among hospitals in the United States, discovered 26 different triage strategies for COVID‐19 pneumonia that 20 of them had utilized Sequential Organ Failure Assessment (SOFA) scoring system models.^13^ SOFA score as an evaluator of organ failure and severity of the disease, was first introduced in 1996 and its functionality has been on the basis of examining the six key organs' functional status: coagulation function, central nervous system, kidney, liver, respiration, and circulation^14^, ^15^ (Supporting Information: Table 1). Although this scoring system was developed to qualify organ failure and not for predicting outcomes, a clear link between mortality and organ dysfunction has been established.^16^ Moreover, some data have supported the accuracy of SOFA score for evaluating the severity and 60‐day mortality of SARS‐CoV2 infected individuals.^17^ However, the hypothesis has remained controversial that the SOFA score has lower accuracy in cases with COVID‐19 pneumonia, albeit an independent risk factor of mortality. That's due to the fact that these individuals are more likely to have significant single‐organ failure and less variability in their SOFA scores. Therefore, we found interest in assessing whether measuring the series of recorded SOFA score alterations could help us refine the clinical outcome of COVID‐19, while being considered an accurate prognostic factor among ICU‐admitted COVID‐19 patients. Furthermore, we have aimed to compare SOFA score measured at different intervals, to other established factors related to COVID‐19 mortality.

## 2 METHODS

### 2.1 Study design

This study was prospectively performed on the COVID‐19‐infected patients who were admitted to the ICU settings of Masih Daneshvari Hospital, Tehran, Iran between March 1, 2020 and March 30, 2022. Masih Daneshvari Hospital was considered as one of the main tertiary patient referral centers during the COVID‐19 pandemic with the remarkable capacity of patient admission (ICU‐beds = 800). All the cases were verified by real‐time‐polymerase chain reaction, and their hospital admission and management were carried out based on the protocols defined by the National Research Institute of Tuberculosis and Lung Diseases (NRITLD) of Iran.^18^ The indications for ICU admission were based on the presence of each of the (1). Respiratory failure which needs invasive/noninvasive ventilation, or (2). Septic shock which needs any dosage of vasopressors; in fact, septic shock is defined as life‐threatening organ dysfunction caused by a dysregulated host response to infection. So the patients with septic shock could be clinically identified by a vasopressor requirement to maintain a mean arterial pressure of 65 mmHg or greater, and the serum lactate level greater than 2 mmol/L (>18 mg/dL) in the absence of hypovolemia.^19^ Moreover, it is worth mentioning that for conducting the current study, the recommendations put forward in the “Guidelines for reporting of statistics for clinical research in urology” were all reviewed and followed for guidance on the proper analysis, reporting, and interpretation of clinical research.^20^ Also, the methodology of this study was checked and revisited based on the STROBE guidelines.^21^

### 2.2 Inclusion and exclusion criteria of the patients

The inclusion criteria of this study were as follows; COVID‐19 patients who were admitted in ICU settings during the first 6 h of referral to emergency department. Based on NIRTLD protocols, all the patients received intravenous methylprednisolone, and additionally, some received Remdesivir with similar standard dosages (methylprednisolone as 1000 mg/day pulse therapy in first 3 days followed by 1 mg/kg in every 12 h for next 10 days, and Remdesivir with loading dosage of 200 mg in 1st day and 100 mg dosage in the next 5 days). Patients who did not undergo the mentioned drugs or received different doses adjusted due to their clinical conditions (such as uncontrolled diabetes or sever renal failure) were excluded from the study. Further, those who were clinically selected to receive anti‐interleukin‐6 (Tocilizumab), Baricitinib, or extracorporeal membrane oxygenation were excluded. Overall, 1057 cases were enrolled in this study. Since this was a prospective cohort, at the end‐point of the study, it was established that patients could all fell into either two groups based on their 28‐day mortality status during ICU admission (765 patients as survived vs. 292 patients as nonsurvived). They had their daily SOFA scores calculated at 5 a.m. morning from ICU admission until discharge/death consecutively. SOFA score was calculated by the skilled emergency medicine, anesthesiology, or internal medicine residents using online medical software “MDCalc” previously authorized for severity scores calculations.^22^ Other data considering demographic, comorbidities, clinical and laboratory characteristics as well as received therapeutics were also compiled.

### 2.3 Data collection

All the mentioned data were prospectively gathered using online collection system forms specifically designed for the hospital research studies. For enhancing the reliability, the data collection process was reassessed by two medical researchers (F. E. T. and F. S. R.) individually and the extracted data were double‐checked.

### 2.4 Definitions

In our assessment, “initial SOFA score” was defined as the SOFA score upon ICU admission; “SOFA score at 48 h” and “SOFA score at 96 h” were defined as SOFA score at 48 and 96 h after ICU admission, respectively; “Δ‐SOFA: 48‐0 h” was considered the subtraction of upon‐hospitalization SOFA score from 48‐h SOFA score; and “Δ‐SOFA: 96‐0 h” was determined as the subtraction of upon‐hospitalization SOFA score from 96‐h SOFA score. “Mean SOFA score” was deemed as the average score of SOFA calculated in the first 4 days of ICU admission.

### 2.5 Statistical analysis

All data were evaluated in terms of being normally disturbed using Shapiro–Wilk test. Continuous and categorical data have been described respectively as mean ± standard deviation and *n* (%). Variables with nonnormal distribution was reported as median (IQR). Mann–Whitney and *χ* ^2^ tests have been employed to juxtapose one group with the other, wherever it suited. Univariate Cox regression analysis for different SOFA statuses and other variables which predicted the risk of mortality among COVID‐19 ICU patients was performed (mean SOFA score, highest SOFA score, initial SOFA score, SOFA score at 48 h, SOFA score at 96 h, Δ‐SOFA score, 48‐0 h a, Δ‐SOFA score, 96‐0 h b, age, length of hospital stay, intubation, diabetes, body mass index [BMI], coronary artery disease [CAD], hypertension). To prevent the overfitting of data, we only included four variables related to COVID‐19 mortality in the multivariate Cox model (CAD, hypertension, diabetes mellitus, and age) to compare with SOFA score trends. The selection of mentioned comorbidities was based on the clinical importance of variables found in recent research^23^, ^24^ as well as data availability of our study. We also designed receiver operating characteristic (ROC) curve for different statuses of the SOFA scores to more effectively represent the predictability of each variable. Two‐sided *α* < ·05 was deemed as the statistical significance. The statistics were all conducted by the IBM SPSS statistics (version: 26.0).

### 2.6 Ethical consideration

This study was reviewed and given approval by the ethics review board of Shahid Beheshti University of Medical Sciences, Tehran, Iran (ethical number: IR. SBMU.1399049). Of note, the present study abided by the principles of the Declaration of Helsinki. All patients and/or their legal guardians were thoroughly delineated about the study protocol. Subsequently, a written informed consent was obtained from all patients and the legal guardians of those who due to their critically ill condition could not provide one.

## 3 RESULTS

### 3.1 Baseline characteristics

All 1057 enrolled patients were split into two groups; the first group entails a total of 765 patients who had survived at least 28 days after study. And the second group is made up of a total of 292 patients who had not survived the first 28 days after study onset. Table 1 shows the initial baseline characteristics of patients divided by study groups. Age as the main demographic feature had a median of 71 years in the survived group, and 74 years in the nonsurvived group; marking a statistically remarkable difference between the two groups (*p* < .001). The sex ratio of cases differed between two groups. Male patients formed 78.3% and 88.0% of the survived group and nonsurvived group, respectively; highlighting that male gender was of statistically significant abundance in the nonsurvived group (*p* < .001). The mean BMI was also recorded to be prominently higher in the nonsurvived group, compared with the survived group (29.4 vs. 27.2; *p* < .001). Patients with no history of prior diseases made up 7.9% and 9% of the cases in the survived group and nonsurvived group, respectively (*p* < .004). Among the different background diseases studied in patients, diabetes mellitus was markedly more prevalent in the nonsurvived group, as against survived group (30.1% vs. 20.6%; *p* < .001). Renal disease, as another background illness, had been reported remarkably more often in the nonsurvived group, compared with the survived group (19.2% vs. 11.5%; *p* < .001). The two study groups had similar prevalence in presence of hypertension, coronary artery disease, cerebrovascular disease, and pulmonary disease. The need for intubation as an important indicator of the patient's well‐being has been reported to be of notable abundance in the nonsurvived group (90.1%), compared with the survived group (66.9%) (*p* < .001). The hospitalization period was also found to last significantly longer in the nonsurvived group than in the survived group (19.2 vs. 16.9 days) (*p* < .001).

**Table 1:** Baseline characteristics of patients with COVID‐19 admitted in intensive care unit.

Characteristics	All patients (*n* = 1057)	28‐day survived (*n* = 765)	28‐day nonsurvived (*n* = 292)	*p* Value
Age, years^a^	72 ± 8.1	71 ± 7.3	74 ± 8.4	<.001
Sex, male	856 (81.0)	599 (78.3)	257 (88.0)	<.001
Active smoker	178 (16.8)	126 (16.5)	52 (17.8)	.60
Body mass index, kg/m^2^	28.0 ± 4.2	27.2 ± 4.3	29.4 ± 3.9	<.001
No disease history	70 (6.6)	61 (7.9)	9 (3.0)	.004
Diabetes mellitus	246 (23.3)	158 (20.6)	88 (30.1)	.001
Coronary artery disease	350 (33.1)	246 (32.1)	104 (35.6)	.28
Cerebrovascular disease	67 (6.3)	46 (6.0)	21 (7.2)	.48
Hypertension	363 (34.3)	270 (35.3)	93 (31.8)	.29
Pulmonary diseases	95 (9.0)	71 (9.3)	24 (8.2)	.58
Renal disease	144 (13.6)	88 (11.5)	56 (19.2)	.001
Malignancy	32 (3.0)	18 (2.3)	14 (4.8)	.38
Intubation	775 (73.3)	512 (66.9)	263 (90.1)	<.001
Length of hospital stay, day	17.5 ± 3.4	16.9 ± 3.2	19.2 ± 4.0	<.001

^a^ Since age has nonnormal distribution, it is reported as median ± IQR.

### 3.2 SOFA score trends as 28‐day mortality predictor

In Table 2 the hazards of mortality for different possible predictive factors have been demonstrated. Based on univariate Cox regression analysis, ICU‐admitted patients with a greater mean SOFA score (1 point higher) were found to be around 3.8 times more probable to expire (*p* < .001). Furthermore, if the highest reported SOFA score of a patient throughout the hospitalization was 1 point higher, that patient was shown to be 2.7 times more likely to die (*p* < .001). Moreover, it was indicated that patients presented with an initial SOFA score of 1 point higher, had a 1.6 times greater chance of death (*p* < .001). The Hazard ratios (HRs) regarding patients' SOFA score reported at 48 and 96 h after admission, were (HR: 1.44, CI: 1.01–2.05) and (HR: 1.31, CI: 1.01–1.69), respectively. It was assessed that the increase in patients' age by 1 year would actually elevate their mortality risk 5.34‐fold (*p* < .001). Also, patients whom hospitalization period had lasted more than 1 day, were 2.2 times more likely to die (*p* < .001). Furthermore, patients who had established a need for intubation, had a 2.3 times greater risk of death. A 1‐point increase in BMI, as another predictive factor, was shown to cause a five‐fold increase in mortality risk of patients (*p* < .001). Diabetes, hypertension, and coronary artery disease have been demonstrated to result in HRs of 2.50, 3.00, and 2.11, respectively. Evaluating multivariate Cox analysis on Table 2, showed that elder age, hypertension, coronary artery diseases, and diabetes mellitus had values of (HR: 3.81 [CI: 1.98–5.21]), (HR: 3.11 [CI: 1.26–3.81]), (HR: 2.82 [CI: 1.51–4.8]), and (HR: 2.45 [CI: 1.36–2.99]), respectively. These all had a stronger correlation to 28‐day mortality compared with mean, highest, and initial SOFA scores. Supporting Information: Figure 1 depicts the ROC curves drawn for eight of the main indices with potential diagnostic roles. The area under the curve (AUC) for each of these eight indices, is a measure of the ability of that classifier to make a distinction. On that account, the higher the AUC gets, the better the diagnostic performance of that index would be. Accordingly, as it is displayed in this figure, our findings showed that the diagnostic factor with the highest AUC is age, therefore it has the best diagnostic ability among all evaluated factors. Other seven diagnostic factors in order of highest to lowest AUCs are mean SOFA scores, highest SOFA score, Δ 0‐96 SOFA score, SOFA score at 96 h, initial SOFA score, Δ 0‐48 SOFA score, and SOFA score at 48 h, with AUCs equal to 0.77, 0.71, 0.64, 0.61, 0.57, 0.55, 0.53, respectively.

**Table 2:** Univariate and multivariate Cox regression analysis of factors predicting risk of mortality among COVID‐19 patients admitted in intensive care unit.

Variables	Univariable, HR (95% CI)	*p* Value	Multivariable, HR (95% CI)	*p* Value
Mean SOFA score	3.82 (2.75–5.31)	<.001	2.28 (2.21–3.51)	<.001
Highest SOFA score	2.70 (1.93–3.78)	<.001	1.24 (0.96–1.59)	.067
Initial SOFA score	1.65 (1.30–2.11)	<.001	1.28 (1.00–1.64)	.095
SOFA score at 48 h	1.44 (1.01–2.05)	.043	–	–
SOFA score at 96 h	1.31 (1.01–1.69)	.038	–	–
Δ‐SOFA score, 48‐0 h^a^	1.17 (0.91–1.50)	.196	–	–
Δ‐SOFA score, 96‐0 h^b^	1.64 (1.18–2.26)	.002	–	–
Age	5.34 (3.86–7.38)	<.001	3.81 (1.98–5.21)	<.001
Intubation	2.37 (1.72–3.27)	<.001	–	–
Body mass index	5.07 (3.40–7.56)	<.001	–	–
Diabetes	2.50 (1.71–3.64)	<.001	2.45 (1.36–2.99)	<.001
Hypertension	3.00 (2.15–4.19)	<.001	3.11 (1.26–3.81)	.03
Coronary artery disease	2.11 (1.66–2.68)	<.001	2.82 (1.51–4.8)	.02

Abbreviations: CI, confidence interval; SOFA, Sequential Organ Failure Assessment.

^a^ Indicates the difference between 48‐h SOFA score and upon hospitalization SOFA score.

^b^ Indicates the difference between 96‐h SOFA score and upon hospitalization SOFA score.

### 3.3 Risk assessment for SOFA score interchanges

**Figure 1:**
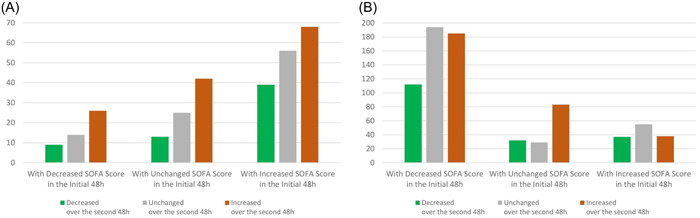
Changes in the SOFA scores of nonsurvivors and survivors from the first 48 h to the second 48 h after admission. (A) Non‐survivors, (B) Survivros. SOFA, Sequential Organ Failure Assessment.

**Figure 2:**
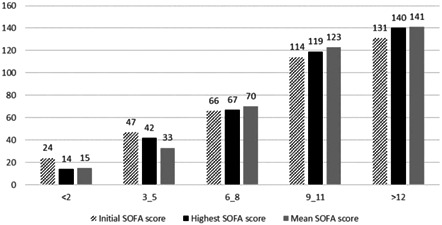
Number of nonsurvived COVID‐19 patients based on the Sequential Organ Failure Assessment (SOFA) score interchanges during the first 96 h of ICU admission.

Table 3 demonstrates the changes of SOFA score at the end of the first and the second 48 h after admission, along with the corresponding mortality rates of patients, based on and categorized by those changes. The overall trend of mortality rates shows that as the changes in SOFA scores within the first 48 h has got higher, at least 50% of mortality occurred regardless of SOFA scores alterations during the second 48 h. Unchanged status of SOFA scores in the first 48 h predicted the mortality rate between 28.8 and 46.29 depending on the alterations of SOFA scores in the second 48 h. Figure 1 visualizes the information in Table 3; it displays how the number of surviving and nonsurviving patients differ when they're grouped based on the changes in SOFA score throughout the first and second 48 h. Of note, SOFA scores depreciation at the first 48 h anticipated 6.73% to 12.32% fatality rate. Figure 2 depicts the number of nonsurvived COVID‐19 cases on the basis of SOFA scores changes in the first 96 h of ICU stay. It could be deduced that overall, a rise‐up in the first 96‐h SOFA scores, comes with an increase in the number of nonsurvived patients. It could also be inferred that, for SOFA scores alterations lower than 6, it is the initial recorded score that has been correlated with the greater number of deaths. While, for SOFA score alterations greater than 8, it is usually the mean recorded score that's been related to higher mortality.

**Table 3:** Changes in the SOFA scores from the first 48 h to second 48 h after admission in intensive care unit in COVID‐19 patients.

First 48 h	Second 48 h	At risk (*n*)	Mortality (*n*)	Mortality rate (%)	Mortality rate in first 96 h (%)
decreased	Decreased	121	9	7.43	6.73–12.32
	Unchanged	208	14	6.73	
	Increased	211	26	12.32	
Unchanged	Decreased	45	13	28.8	28.8–46.29
	Unchanged	54	25	46.29	
	Increased	125	42	33.6	
Increased	Decreased	76	39	51.31	50.45–64.15
	Unchanged	111	56	50.45	
	Increased	106	68	64.15	

*Note*: First 48 h = SOFA score Day 2 of ICU admission_ SOFA score upon ICU admission; Second 48 h = SOFA score Day 4 of ICU admission_ SOFA score Day 2 of ICU admission.

Abbreviation: SOFA, Sequential Organ Failure Assessment.

## 4 DISCUSSION

‏The incentive to the present research was to evaluate whether the SOFA score would help to clarify the clinical outcomes of COVID‐19, while also investigating whether SOFA score could be considered an accurate predictive element among ICU‐admitted COVID‐19 patients. While there have been several studies previously performed, that assessed the accuracy of SOFA score predicting ability in COVID‐19 mortality, they had four main drawbacks. These drawbacks made us design this study: first, they were merely focused on SOFA score upon admission while not being consecutively assessed^16^, ^25^, ^26^, ^27^; herein we have moved a step further by assessing the mean SOFA score as a more reliable 28‐day mortality predictor during ICU‐stay instead of only initial SOFA score (AUC for initial SOFA = 0.57 vs. mean SOFA = 0.77), as well as the first 96 h serial followup after ICU admission. Second, the paradoxical obtained results which some studies had adequate discriminating accuracy for SOFA score as COVID‐19 mortality risk factor and some contrarily did not support.^28^ Third, all patients in this study admitted to the ICUs required mechanical ventilation (noninvasive or invasive ventilation), which was indicative of a critical clinical status for COVID‐19 pneumonia in all cases. Meanwhile, such patients in other studies did not exhibit severe respiratory failure and sometimes even were not admitted to ICU settings, which therefore could be potentially associated to better survival. And fourth, we had a relatively large study population (1057 patients) and we conducted this study prospectively; compared with retrospective studies previously performed.^16^ Until now, no high‐yield systematic review analysis has been reported on the SOFA score's trends accuracy and its predictive ability in detecting COVID‐19 severity. This scoring system was developed to quantify organ failure degrees in patients with severe sepsis, and it has been since well‐established for use in ICU patients globally.^29^ The SOFA scoring system has been commonly employed to foretell the clinical aftermath of critically ill cases. For instance, it could estimate the possibility of death in cases with hematologic malignancies and chronic liver failure, mainly because critically ill patients almost always have multiple organ failures.^30^, ^31^ SARS‐CoV‐2 infection, according to Gupta et al., can cause significant lung injury as well as damage to the skin, blood system, endocrine system, neurological system, kidney, liver, and heart, which could all lead to skin lesions, hyperglycemic state, gastrointestinal manifestations, thrombotic state, acute coronary syndrome, and arrhythmia.^4^ Even though we found that serial SOFA score could be a self‐reliant mortality risk factor for SARS‐CoV‐2 infection, it was still less accurate compared with other established COVID‐19 mortality risk factors based on both multivariate analysis (age: HR: 3.81 [95% CI: 1.98–5.21, *p* < .001] vs. mean SOFA score (HR: 2.28 [95% CI: 2.21–3.51, *p* < .001]) and area under ROC (AUC for age = 0.81 vs. AUC for mean SOFA score = 0.77). These results are in accordance with a previous study on 675 critically ill COVID‐19 patients, prepared by Raschke and his colleagues. They confirmed that the SOFA score is not an accurate index to be employed as a ventilator triage method for COVID‐19 cases compared with age (age‐AUROC = 0.66 vs. SOFA‐AUROC = 0.59).^28^ This could be reasoned by the clinical fact that patients with severe COVID‐19 infection were mostly expired due to only three of six organ failures (hepatic, renal, and especially respiratory system). These three organ functions are evaluated in the SOFA criteria but patients may not have severe dysfunction in other organ systems. On the other hand, the AUROC for SOFA score obtained in this study was less than AUROC of previously performed studies.^19^, ^32^ The mentioned studies showed substantial discriminating efficiency for SOFA score in determining survival rate of ICU patients with sepsis, as well as an AUROC of 0.74–0.75. This pointed to the fact that SOFA score accuracy for mortality prediction among COVID‐19 patients does not seem to be as useful as those who are ICU‐admitted for other reasons.^19^, ^32^ Therefore, one possible solution is to define another scoring system that measures only degrees of organ failure that are strongly associated with COVID‐19 such as oxygen saturation while also taking into account other well‐known factors including age, diabetes mellitus, hypertension, or CAD. Such risk factors should show high odds ratios in predicting mortality. Of note, a recent study by Guarino et al mentioned new scale of ICU admission prognosis score named modified quick SOFA score (Mq‐SOFA) to evaluate in‐hospital survival for COVID‐19 cases in Italy.^33^ They defined Mq‐SOFA based on four parameters: SpO2/FiO2 ratio, respiratory rate ≥22/min, systolic blood pressure ≤100 mmHg, and considerable disorientation or confusion. By doing so, they found that Mq‐SOFA could reliably predict both in‐hospital mortality and 30‐day mortality with high levels of sensitivity (58.1% and 84.2%, respectively), and diagnostic accuracy (78.3% and 77.6%, respectively).

The present study has several limitations. First due to the missing data on electronic health records of patients we had to exclude those who had incomplete clinical data, so the initial study population had to be diminished leaving us with a smaller study sample. Moreover, we were not able to reveal the precise extent of missing data in our SOFA score reports, due to some practical complications in the hospital, where we conducted the study. Second, one should notice the absence of so‐called “soft” clinical data in this study, could potentially play an important role in understanding the mortality in COVID‐19 patients. In fact, some large prospective cohorts have discussed that such data, that is, frailty, daily‐life activities, could plausibly influence mortality rate of COVID‐19, particularly in elderly patients.^34^, ^35^ Since the data of our study lacks such information regarding activity of daily life or frailty, we could have overlooked its potential role in impacting the 28‐day mortality in patients; so future studies are needed to shed light on the matter.

## 5 CONCLUSION

In this perspective and single‐center study we found that the mean SOFA score in first 96 h of COVID‐19‐related ICU admission could predict 28‐days in hospital mortality reliably and stronger than SOFA score upon admission, highest SOFA score during hospital stays, and other SOFA score time interchanges. However, the prognostic value of mean SOFA score as independent risk factor of mortality was not statically as strong as established COVID‐19 fatality risk factors (age, diabetes mellitus, hypertension, and CAD). Furthermore, increased SOFA score in first 48 h of ICU stay prognosticate of at least 50% fatality regardless of initial SOFA score.

## AUTHOR CONTRIBUTIONS

**Farzad Esmaeili Tarki**: conceptualization; formal analysis; investigation; methodology; writing—original draft; writing—review & editing. **Siamak Afaghi**: conceptualization; formal analysis; investigation; methodology; project administration; resources; software; supervision; validation; visualization; writing—original draft; writing—review & editing. **Fatemeh Sadat Rahimi**: conceptualization; data curation; methodology; writing—original draft; writing—review & editing. **Arda Kiani**: conceptualization; supervision; writing—original draft. **Mohammad Varahram**: conceptualization; data curation; formal analysis. **Atefeh Abedini**: project administration; supervision; validation; writing–original draft.

## CONFLICTS OF INTEREST STATEMENT

The authors declare no conflict of interest.

## TRANSPARENCY STATEMENT

The lead author Atefeh Abedini affirms that this manuscript is an honest, accurate, and transparent account of the study being reported; that no important aspects of the study have been omitted; and that any discrepancies from the study as planned (and, if relevant, registered) have been explained.

## Supporting information

Supplementary information.

Supplementary information.

Supplementary information.

## Data Availability

The data that support the findings of this study are all available for interested readers on request from the corresponding author.
