# Supplementary figures and images for: Serial SOFA‐score trends in ICU‐admitted COVID‐19 patients as predictor of 28‐day mortality: A prospective cohort study
Source: Health Sci Rep. 2023 May 2;6(5):e1116. doi: 10.1002/hsr2.1116 (PMC10154817; doi:10.1002/hsr2.1116)

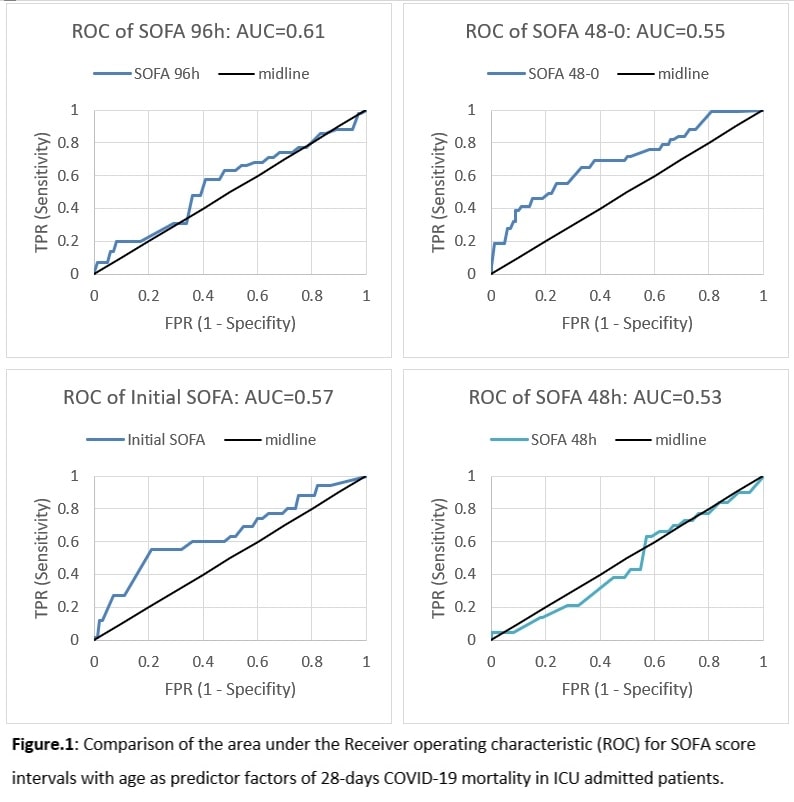

Supplement: Supplementary file 1 — Supplementary information. [file HSR2-6-e1116-s001.jpg]

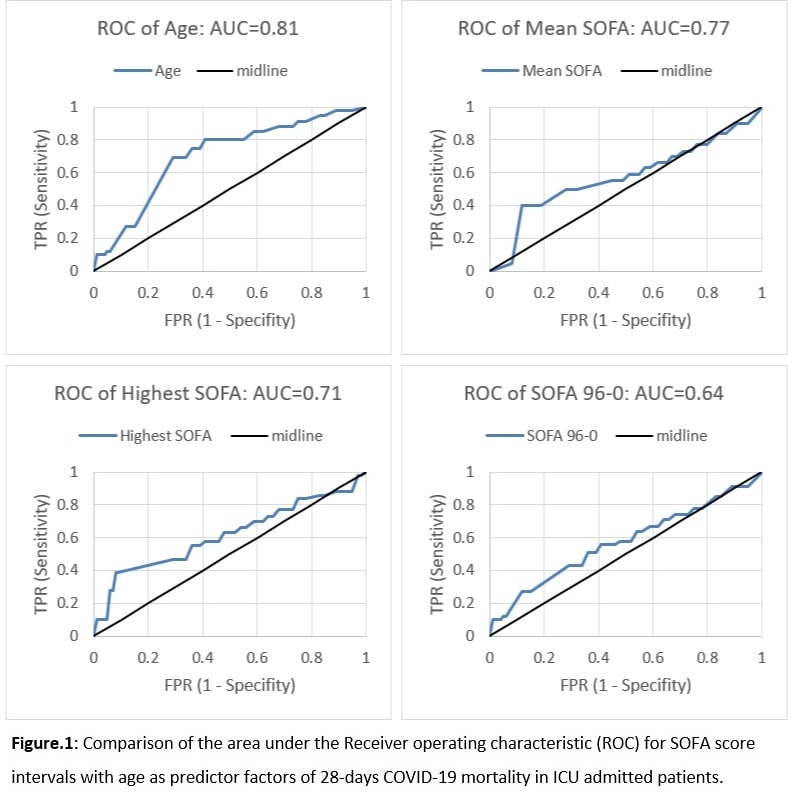

Supplement: Supplementary file 2 — Supplementary information. [file HSR2-6-e1116-s003.jpg]
