# Supplementary material for: Serial SOFA‐score trends in ICU‐admitted COVID‐19 patients as predictor of 28‐day mortality: A prospective cohort study
Source: Health Sci Rep. 2023 May 2;6(5):e1116. doi: 10.1002/hsr2.1116 (PMC10154817; doi:10.1002/hsr2.1116)
Supplement: Supplementary file 3 — Supplementary information. [file HSR2-6-e1116-s002.docx]

| **Supplementary Table.1:** The Sequential Organ Failure Assessment (SOFA) score^1^ . | | | | | |
| --- | --- | --- | --- | --- | --- |
| Parameter | SOFA score | | | | |
|  | 0 | 1 | 2 | 3 | 4 |
| Respiratory: PaO_2_/FiO_2_ (mmHg) | >400 | ≤400 | ≤300 | ≤200 ^a^ | ≤100 ^a^ |
| Cardiovascular: Hypotension | No hypotension | MAP <70mmHg | Dopamine ≤5  Or  Dobutamine (any dose)^2^ | Dopamine >5  Or  Epinephrine ≤0.1  Or  Norepinephrine≤0.1^b^ | Dopamine >15  Or  Epinephrine >0.1  Or  Norepinephrine>0.1^b^ |
| Central nervous system: GCS | 15 | 13-14 | 10-12 | 6-9 | <6 |
| Bilirubin (mg/dl) | <1.2 | 1.2-1.9 | 2.0-5.9 | 6.0-11.9 | >12.0 |
| Coagulation: Platelets (×10^3^/mm^3^) | >150 | ≤150 | ≤100 | ≤50 | ≤20 |
| Renal: Creatinine (mg/dl)  Or urine output | <1.2 | 1.2-1.9 | 2.0-3.4 | 3.5-4.9  <500 mL/day | >5.0  <200 mL/day |
| ^a^: With respiratory support.  ^b^: Adrenergic agents administered for at least 1 hour (doses given are in mcg/kg per min).  MAP=Mean Arterial Pressure; GCS=Glasgow Coma Scale  1. Lambden S, Laterre PF, Levy MM, Francois B. The SOFA score-development, utility and challenges of accurate assessment in clinical trials. Crit Care. 2019;23(1):374. | | | | | |
